# Seroprevalence of human brucellosis and molecular characteristics of *Brucella* strains in Inner Mongolia Autonomous region of China, from 2012 to 2016

**DOI:** 10.1080/22221751.2020.1720528

**Published:** 2020-01-30

**Authors:** Zhi-guo Liu, Miao Wang, Na Ta, Meng-gang Fang, Jing-chuan Mi, Rui-ping Yu, Yao Luo, Xiaoan Cao, Zhen-jun Li

**Affiliations:** a State Key Laboratory for Infectious Disease Prevention and Control, National Institute for Communicable Disease Control and Prevention, Chinese Center for Disease Control and Prevention, Beijing, People’s Republic of China; b Inner Mongolia Autonomous Region Comprehensive Center for Disease Control and Prevention, Huhhot, People’s Republic of China; c Ulanqab Centre for Endemic Disease Prevention and Control, Jining, Inner Mongolia; d Farmer School of Business, Miami University, Oxford, OH, USA; e State Key Laboratory of Veterinary Etiological Biology, Lanzhou Veterinary Research Institute, Chinese Academy of Agricultural Sciences, Lanzhou, People’s Republic of China

**Keywords:** Serology, *Brucella melitensis*, MLVA, molecular epidemiology, Inner Mongolia

## Abstract

In the present study, a total of 1102304 serum samples were collected to detected human brucellosis between the years 2012 and 2016 in Inner Mongolia. Overall, an average of 3.79% anti-Brucella positive in Inner Mongolia was presented but the range of positive rates were among 0.90 to 7.07% in 12 regions. Seroprevalence of human brucellosis increased gradually from 2012 to 2016. However, the incidence rate of human brucellosis showed a declining trend. One hundred and seven Brucella strains were isolated and identified as *B. melitensis* species, and *B. melitensis* biovar 3 was the predominant biovar. MLVA-11 genotypes 116 was predominant and had crucial epidemiology to the human population. All 107 strains tested were sorted into 75 MLVA-16 genotypes, with 54 single genotypes representing unique isolates. This result revealed that these Brucellosis cases had epidemiologically unrelated and sporadic characteristics. The remaining 21 shared genotypes among two to four strains, confirming the occurrence of cross-infection and multiple outbreaks. Extensive genotype-events were observed between strains from this study and Kazakhstan, Mongolia, and Turkey, these countries were key members of the grassland silk road. Long-time trade in small ruminants (sheep) in these countries has possibly promoted the spread of *Brucella spp*. in these regions.

## Introduction

Brucellosis is a highly contagious zoonosis that can occur by direct contact with infected animals or through the indirect consumption of contaminated dairy products [1]. The causative agents of brucellosis belong to the genus *Brucella*, which are nonmotile, Gram-negative, and facultative intracellular pathogens belonging to the class α-proteobacteria [2]. The genus is represented by 12 known species [3]; among these, *Brucella melitensis*, *Brucella abortus*, and *Brucella suis* have a particularly critical and extensive impact on human health and the development of animal husbandry [4,5]. Brucellosis is a common zoonosis in northern China [6], However, the Inner Mongolia Autonomous Region is an area in China of known endemicity, with the highest incidence rate, accounting for approximately 40% of reported cases in the country during 2011–2016 [7,8]. The incidence and epidemiology of brucellosis in this region represent the characteristics of this disease in China. *B. melitensis* has been the predominant species associated with human outbreaks and sporadic cases in this province; *B. abortus and B. suis* have also been associated with sporadic brucellosis cases [9]. Genotyping by multilocus sequence typing (MLST) of isolates has shown that the sequence types of strains from high-incidence stages differed from those at the low-incidence stage in Inner Mongolia [10]. Available and comprehensive epidemiological characterization of human brucellosis is lacking in the Inner Mongolia area. The seroprevalence investigation, identification, and molecular characterization of human brucellosis are the cornerstones for understanding the epidemiology of the disease in a region and implementing adequate strategies to control this important zoonosis [11]. Previous studies have confirmed that the multiple-locus variable-number tandem-repeat analysis (MLVA) scheme is now widely used and often allows for the fine-scale resolution of closely related isolates [12,13]. This assay generated both a regional and global context for genetic characterization of *Brucella*. The seroepidemiology of human brucellosis in Inner Mongolia from 2012 to 2016 was investigated and analyzed. We also genetically characterized 107 human brucellosis isolates from cases between 2012 and 2016 in Inner Mongolia. Thus, the main purpose of this study was to elucidate the epidemiological characterization of human brucellosis and molecular epidemiology links of *B. melitensis* strains obtained from the Inner Mongolia Autonomous Region.

## Materials and methods

### Ethics statement

This research was carried out according to the principles of the Declaration of Helsinki. The study protocol was approved by the Ethics Committees of the National Institute for Communicable Disease Control and Prevention, Chinese Center for Disease Control and Prevention. Informed consent was obtained from all patients prior to diagnosis and patient data were anonymized. *Brucella* spp*.* was isolated from patient blood samples following confirmation of consent.

### Collection of serum samples and serology detecting

**Figure 1.**
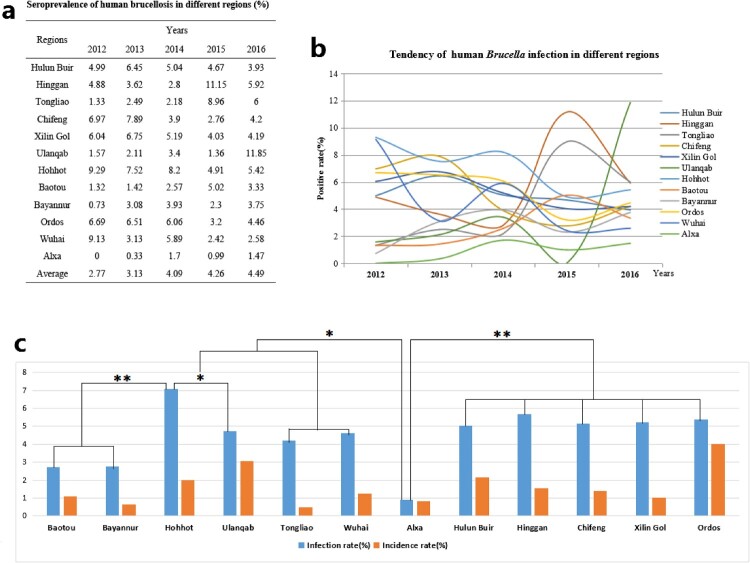
Seroprevalence characteristics of human brucellosis in different regions of Inner Mongolia, China, from 2012 to 2016, and * *P* < 0.05; ** *P* < 0.01 are used to indicate statistical significance levels.

From 2012 to 2016, blood samples were collected from patients residing in the Inner Mongolia Autonomous Region of China. During this period, a total of 1102304 serum samples were collected for serological testing in accordance with the “Diagnostic standard for brucellosis” (WS269-2007) [14,15]. These serum samples were collected from 12 regions, including Hulun Buir (*n* = 104504), Hinggan (*n* = 70,413), Tongliao (*n* = 23261), Chifeng (*n* = 69188), Xilin Gol (*n* = 116516), Ulanqab (*n* = 117, 051), Huhhot (*n* = 37532), Baotou (*n* = 166727), Bayannur (*n* = 307707), Ordos (*n* = 55936), Wuhai (*n* = 22302), and Alxa (*n* = 7603) (Figure 1, Table 1 and Table S2). All serum samples were transported to the laboratory, where they were stored at −20 °C until processing by isolation or detection using the Rose Bengal Plate Test (RBPT) and the positive serum sample results were confirmed using the Standard Agglutination Test (SAT) [16]. All reagents were procured from Qingdao Zhongchuang Biotechnology Co., Ltd.

**Table 1.** Sample size and test data in different regions from 2012 to 2016 years.

Regions	2012			2013			2014			2015			2016		
	Size	*P* size	M size	Size	*P* size	M size	Size	*P* size	M size	Size	*P* size	M size	Size	*P* size	M size
Hulun Buir	5475	273	176	4413	290	182	7711	389	205	48839	2283	302	38066	1496	110
Hinggan	14582	711	529	11159	354	216	25958	726	398	9756	1088	50	8958	530	474
Tongliao	903	12	0	5049	83	4	1009	22	11	8502	762	87	7798	468	68
Chifeng	5131	337	253	5426	428	256	14162	553	423	23440	646	0	21029	883	210
Xilin Gol	5015	303	130	57249	341	51	8184	587	74	15574	628	144	30494	1280	161
Ulanqab	12937	203	194	72030	226	198	12629	430	402	11360	461	168	8095	959	801
Hohhot	4694	436	168	4497	332	91	13057	1071	180	7757	378	153	7527	408	80
Baotou	82721	1096	185	9774	828	201	43371	1116	455	18924	950	453	11937	397	328
Bayannur	24067	697	415	58440	2217	310	126081	4894	2148	57132	1335	354	41987	1574	475
Ordos	1987	133	130	10669	727	639	17516	1062	883	13963	446	427	11801	526	505
Wuhai	3383	309	95	3326	1793	381	12758	751	141	1325	32	15	1510	39	9
Alxa	1810	0	0	1817	6	6	1000	17	16	1003	10	10	1973	29	23
Total	162705	4510	2275	243849	7625	2486	283436	11618	5336	221139	9423	2341	191175	8589	3244

Note: Size sample = number collection, *P* size = positive number, M size = morbidity number.

### Isolation of *Brucella spp.* and identification

Blood samples from 1460 patients with brucellosis who presented with fever (antibody titre of SAT ≥1:200) were collected for pathogen isolation. These blood samples were collected from 12 cities, including Hulun Buir (*n* = 567), Hinggan (*n* = 40), Tongliao (*n* = 57), Chifeng (*n* = 50), Xilin Gol (*n* = 47), Ulanqab (*n* = 230), Huhhot (*n* = 280), Baotou (*n* = 12), Bayannur (*n* = 97), Ordos (*n* = 36), Wuhai (*n* = 14) and Alxa (*n* = 30). The isolated experiments were performed according to the standard diagnosis approach for brucellosis; 5–10 mL of fresh blood were collected from patients with brucellosis and injected into a double blood culture bottle (biomerieux.com.cn) under sterile conditions, and cultured for at least 30 days in a CO_2_ incubator. Negative culture bottles were autoclaved at 121°C, for 30 min. *Brucella* strains were biotyped using standard procedures [14]. The *B. melitensis* 16M, *B. abortus* 544, and *B. suis* 1330 reference strains were used as the control strains. Species-level identification was performed using *B. abortus*, *B. melitensis*, *B. ovis*, and *B. suis* PCR (AMOS-PCR) [17]. DNA was extracted with a nucleic acid automatic extraction system (LLXBIO China Ltd., China) using a single loop of fresh *Brucella* cells that were grown for 48 h on *Brucella* agar.

### *Brucella* MLVA-16 genotyping scheme

MLVA was performed as described previously [18]. The PCR products for 16 loci were denatured and resolved by capillary electrophoresis on an ABI Prism 3130 automated fluorescent capillary DNA sequencer (Applied Biosystems, Foster City, California, USA). Fragments were sized following a comparison with a ROX (carboxy-X-rhodamine)-labeled molecular ladder (MapMaker 1000; Bioventures Inc., Murfreesboro, TN, USA) and Gene Mapper software version 4.0 (Applied Biosystems). The fragment sizes were subsequently converted to a number of repeat units using a published allele numbering system [19].

### Analysis of data

Throughout the process, Microsoft Excel was used for data cleaning. Data were analyzed using SPSS19 and JMP Pro 14. Pearson’s correlation coefficient was used to explain the relationship between infections and morbidity; *P*-values < 0.05 were considered significantly different. Polymorphisms at each locus were quantified using the Hunter–Gaston Diversity Index (HGDI) [20]. The resultant genotypes were compared using the web-based *Brucella* 2016 MLVA database (V1.4.0) (http://microbesgenotyping.i2bc.paris-saclay.fr/databases). BioNumerics version 5.1 software (Applied Maths, Belgium) was used to analyze the data obtained from the MLVA-16 assay. Both the categorical coefficient and the unweighted pair group methods were used for clustering analysis. Moreover, 2180 *B. melitensis* strains from 30 different National (Region) regions were used to construct the minimum spanning tree (MST) based on the MLVA-16 data. This was used to explore the genetic relationships among these strains at a global level.

## Results

### Human brucellosis in Inner Mongolia from 2012 to 2016

**Figure 2.**
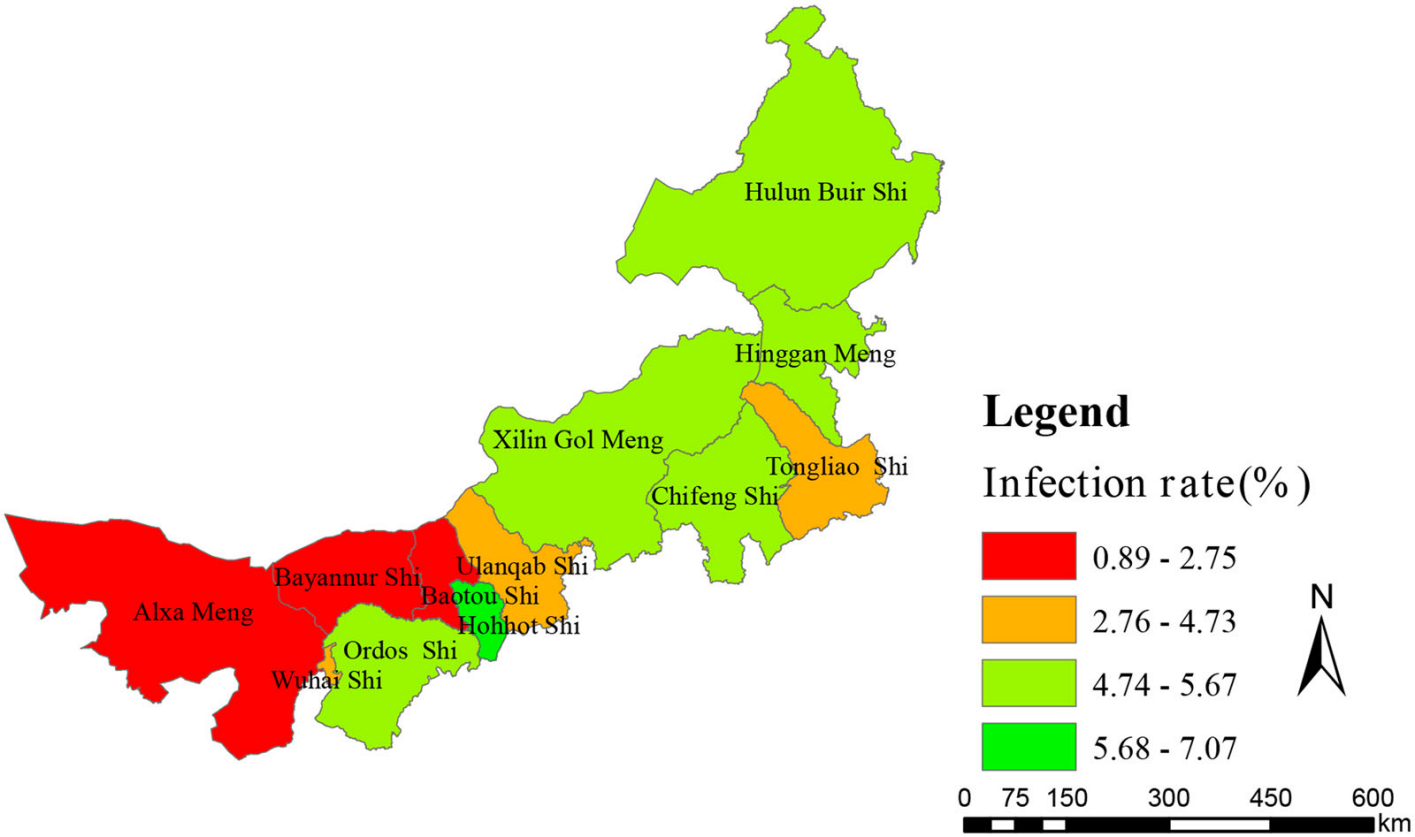
Infection rate profiles of human brucellosis in different regions of Inner Mongolia, China, from 2012 to 2016.

A total of 1102304 serum samples were collected and tested. Of these, 41765 (3.79%) were anti-*Brucella* positive (Table 1). The yearly positive rates from 2012 to 2016 were 2.77%, 3.13%, 4.09%, 4.26% and 4.49%, respectively. From 2012 to 2016, the average positive rates in each region ranged from 0.90 to 7.07% in 12 regions. In addition, the yearly prevalence rates were ranged from 0 to 11.85% (Figures 1a, b). Although the emergence of this disease increased gradually, visibly differences of Brucella-infected patients were observed form some regions in different years, indicating that outbreaks of human brucellosis occurred in these places (Figures 1a, b). From a geographical perspective, human brucellosis showed regional differences (Figure 1c). Generally, human brucellosis was more prevalent in eastern than western areas of Inner Mongolia. However, Hohhot, a capital city of Inner Mongolia, had a greater number of *Brucella-*infected people (Figure 2).

### Analysis of infection and incidence of human brucellosis

**Figure 3.**
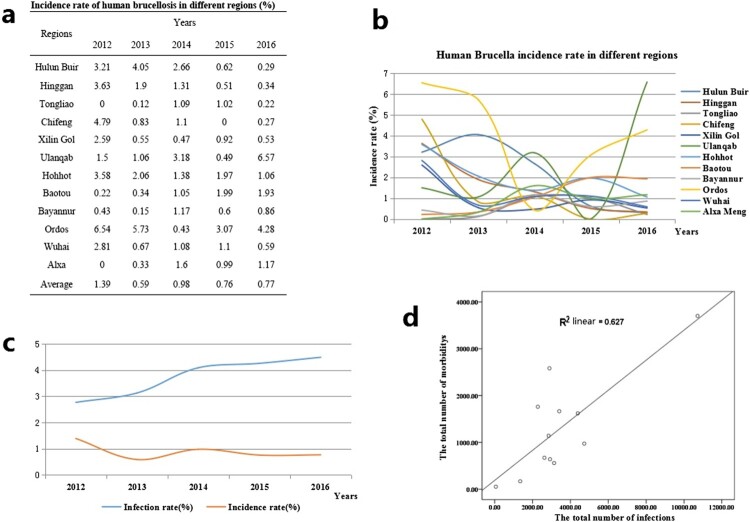
Incidence rate characteristics of human brucellosis in different regions of Inner Mongolia, China, from 2012 to 2016.

Of a total of 1102304 people, 15628 (1.42%) presented morbidity. The antibody titre of the patients was ≥1:50 and their clinical manifestations included chills, weakness, sweating, headaches, as well as muscle and joint pain. The annual incidence rates from 2012 to 2016 were 1.39%, 0.59%, 0.98%, 0.76% and 0.77%, respectively. From 2012 to 2016, the average incidence rate in each region ranged from 0.49 (490/100 000) to 4.01% (4 010/100 000) in the 12 regions. In addition, the annual incidence rates ranged between 0 and 6.57% in these 12 regions (Figure 3a, b). However, from 2012 to 2016, incidence rates were relatively stable while infection rates presented a rising trend. However, the incidence rate and the number of infected people appeared to have a significant linear correlation (*r* = 0.627, *P* < 0.01) (Figure 3c, d). From a regional perspective, the results showed a strong positive relationship between the infection and incidence rates in Hulun Buir, Hinggan, Chifeng, Ulanqab, Ordos, and Alxa (positive relationship index between 1.506 and 0.88), with a weak positive relationship in Tongliao, Xilin Gol, Huhhot, Bayannur, and Wuhai (positive relationship index between 0.141 and 0.206), but this index was unclear in Baotou. The results also showed that there were noticeable differences between the morbidity number as a percentage of the number of infections. The percentage of morbidity was extremely high (above 77%) in Ulanqab, Ordos, and Alxa, but was low (below 22%) in Wuhai, Hulun Buir, Tongliao, and Xilin Gol.

### Identification and distribution of *B. melitensis*

**Figure 4.**
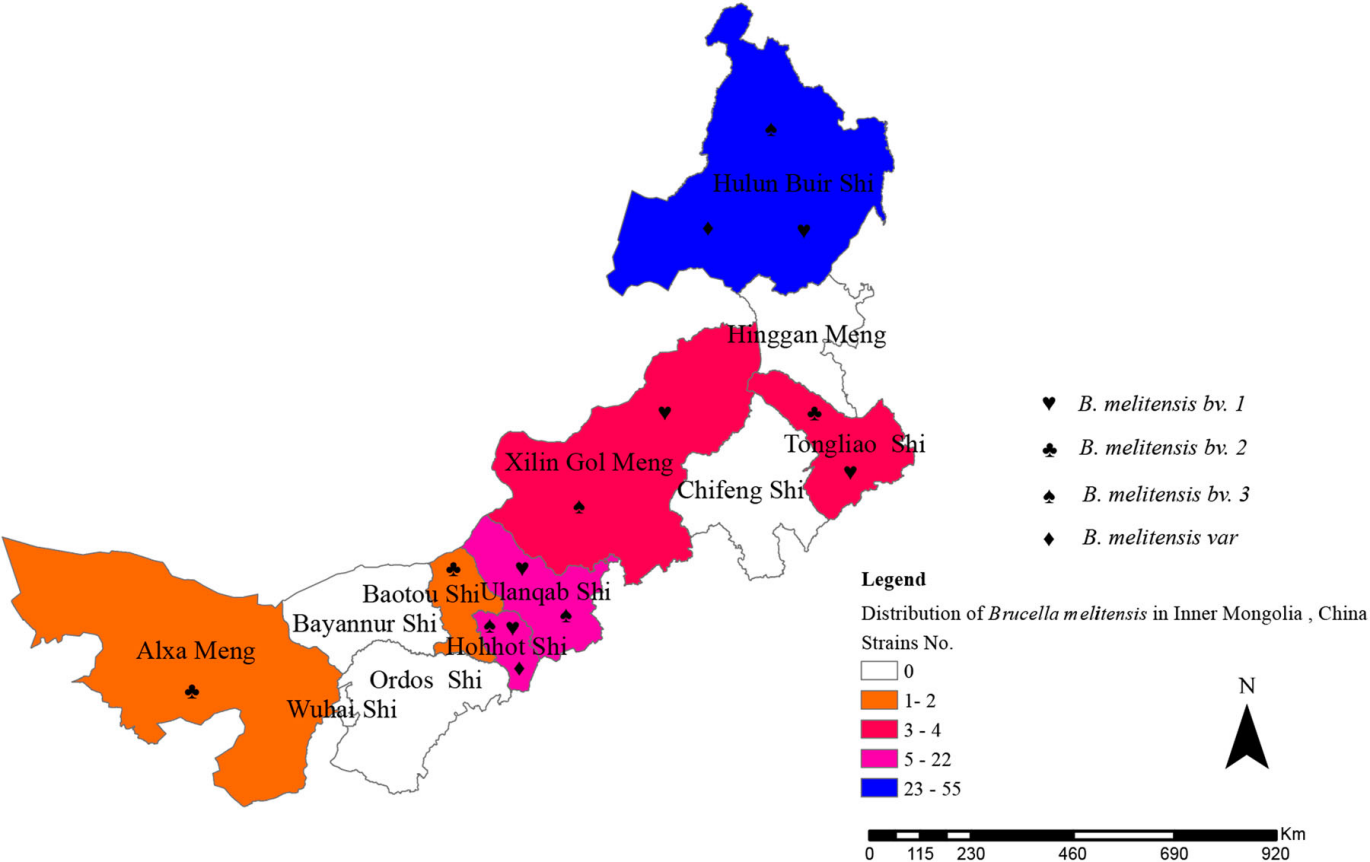
Geographic distribution characteristics of *B. melitensis* in difference regions, Inner Mongolia, China, from 2012 to 2016.

**Figure 5.**
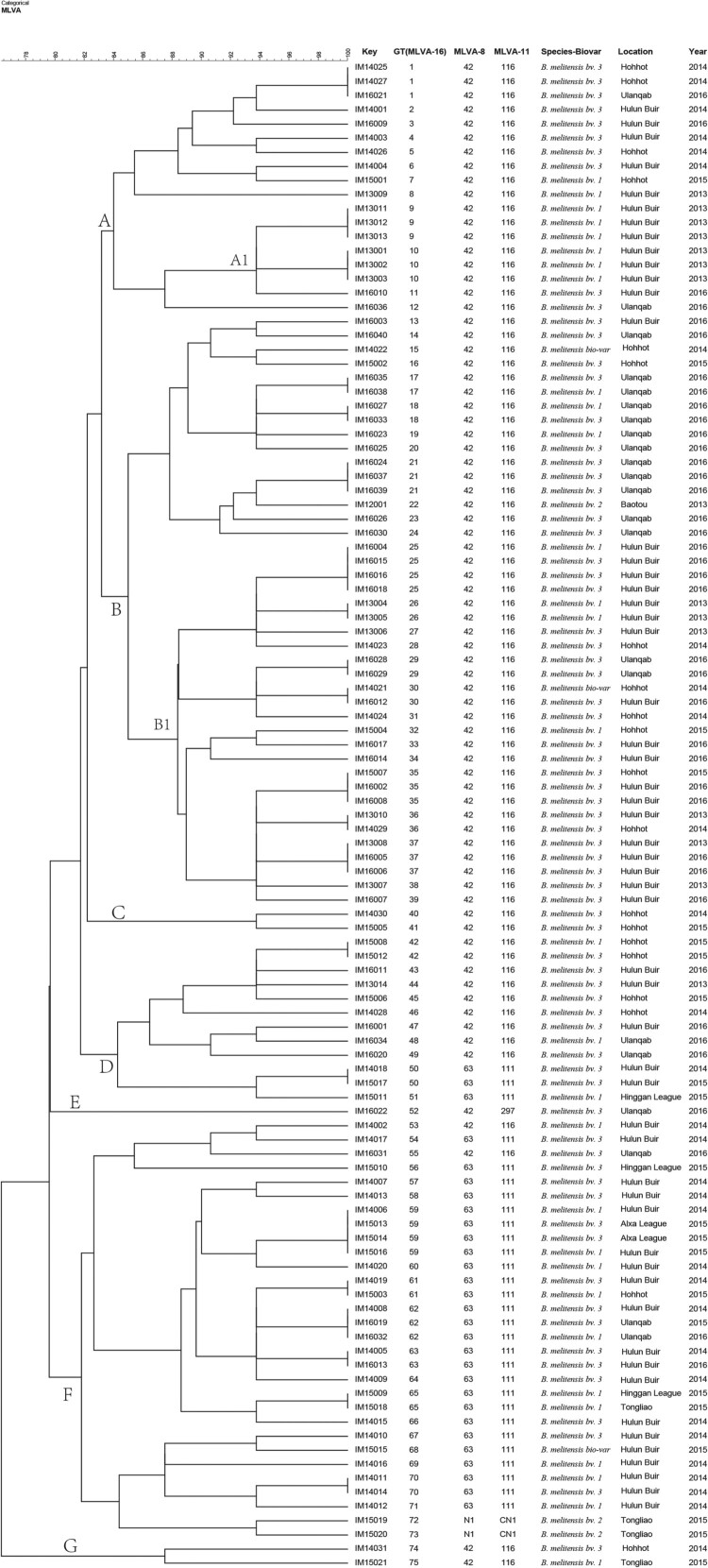
Dendrogram based on the multilocus variable-number tandem-repeat analysis (MLVA)-16 genotyping assay (unweighted pair group method with arithmetic mean (UPGMA) method), showing relationships between 107 *B. melitensis* isolates. Columns show identification numbers (Key), MLVA-16 genotypes (GT), panel 1 genotypes and MLVA-11 (panels 1 and 2A) genotypes, species biovar and the year in which strains were isolated.

All 107 *Brucella* strains were identified as *B. melitensis* using the classical biotyping procedure*.* Colony morphology, staining, growth characteristics, and slide agglutination with monospecific anti-*Brucella* sera were used to characterize all isolates (Table 2)*.* Globally, biotyping identified 30 strains as *B. melitensis* biovar 1, two strains as *B. melitensis* biovar 2, 71 as *B. melitensis* biovar 3, and three as *B. melitensis* bio-Var, of which *B. melitensis bv. 3* was the most circulated species-biovar in the examined region. These strains were further examined by AMOS-PCR, and all strains were obtained as 731 bp amplification products. The AMOS-PCR identified results were consistent with the classical biotyping approach. Overall, 107 *B. melitensis* strains were observed in seven areas of the Inner Mongolia Autonomous Region, including Baotou (*n* = 1), Huhhot (*n* = 18), Hulun Buir (*n* = 55), Hinggan League (*n* = 3), Alxa League (*n* = 2), Tongliao (*n* = 4), and Ulanqab (*n* = 22) (Figure 4 and Table S1). The strains were predominantly distributed in the eastern and middle areas of the Inner Mongolia Autonomous Region, particularly Ulanqab (22 isolates) and Hulun Buir (55 isolates) areas (Table 3)*.* The distribution of strains was consistent with the epidemiological characteristics (Figure 5) of these regions.

**Table 2.** Classical biotyping assay for identified isolates from Inner Mongolia Autonomous Region.

Strain No.	Growth characteristics				Monospecific sera			Phage lysis testing			Interpretation
	CO_2_ requested	H_2_S	BF	TH	A	M	R	Tb	BK_2_	Wb	
*B*. *abortus* 544 (1)	+	−	+	−	+	−	−	CL	CL	NL	*B*. *abortus bv.1*
*B*. *melitensis* 16M (1)	−	−	+	+	−	+	−	NL	CL	NL	*B*. *melitensis bv. 1*
*B*. *suis* 1330 (1)	−	+	−	+	+	−	−	NL	CL	CL	*B*. *suis bv. 1*
30	−	−	+	+	−	+	−	NL	CL	NL	*B*. *melitensis bv. 1*
2	−	−	+	+	+	−	−	NL	CL	NL	*B*. *melitensis bv. 2*
71	−	−	+	+	+	+	−	NL	CL	NL	*B*. *melitensis bv. 3*
3	−	−	+	+	+	+	−	NL	CL	NL	*B*. *melitensis bv-var*

**Table 3.** Strain key, species-biovar, number, and location used in this study.

Key (code)	*Species/biovar*	Numbers	Total	Location
IM15013∼14	*B. melitensis bv. 3*	2	2	Alxa League
IM12001	*B. melitensis bv. 2*	1	1	Baotou
IM15009∼11	*B. melitensis bv. 1*	2	3	Hinggan League
	*B. melitensis bv. 3*	1		
IM15001∼08, 012, IM14021∼31	*B. melitensis bv. 1*	4	20	Huhhot
	*B. melitensis bio-var*	2		
	*B. melitensis bv. 3*	14		
IM13001∼14, IM14001∼20, IM15015∼17, IM16001∼18	*B. melitensis bv. 1*	17	55	Hulun Buir
	*B. melitensis bio-var*	1		
	*B. melitensis bv. 3*	37		
IM15018∼21	*B. melitensis bv. 1*	2	4	Tongliao
	*B. melitensis bv. 2*	2		
IM16019-40	*B. melitensis bv. 1*	5	22	Ulanqab
	*B. melitensis bv. 3*	17		

### Allele types and genetic diversity of *B. melitensis* strains

In this study, the results of the *B. melitensis* strain diversity analysis confirmed the high discriminatory power of the MLVA-16 assay; the polymorphism index was 0.992 according to the HGDI, as compared to 0.434 and 0.447 for the MLVA-8 and MLVA-11 panels, respectively (Table 4). The HGDI in panel 1 was the highest (0.426) for bruce43. The bruce06, bruce08, bruce11, bruce12, bruce42, bruce45, and bruce55 loci of panel 1, and the bruce18 and bruce19 loci of panel 2A showed only one allele (HGDI = 0). In contrast, the greatest variability was observed in panel 2B (HGDIs = 0.988), particularly the bruce04, bruce16, and bruce30 loci, of which the bruce16 locus exhibited the highest diversity (HGDI = 0.843) (Table 4).

**Table 4.** Hunter–Gaston diversity and allele types of *B. melitensis* in Inner Mongolia, China.

Locus	Diversity index	Confidence interval	K	Max (pi)
Bru06	0.000	0.000–0.065	1	1.000
Bru08	0.000	0.000–0.065	1	1.000
Bru11	0.000	0.000–0.065	1	1.000
Bru12	0.000	0.000–0.065	1	1.000
Bru42	0.000	0.000–0.065	1	1.000
Bru43	0.426	0.388–0.464	3	0.710
Bru45	0.000	0.000–0.065	1	1.000
Bru55	0.000	0.000–0.065	1	1.000
Panel 1	0.434	0.397–0.471	3	0.701
Bru18	0.000	0.000–0.065	1	1.000
Bru19	0.019	0.002–0.036	2	0.991
Bru21	0.000	0.000–0.065	1	1.000
MLVA-11	0.447	0.410–0.484	4	0.692
Bru04	0.801	0.767–0.835	9	0.299
Bru07	0.037	0.014–0.060	2	0.981
Bru09	0.019	0.002–0.036	2	0.991
Bru16	0.843	0.831–0.855	8	0.252
Bru30	0.696	0.666–0.725	6	0.477
Panel 2B	0.988	0.985–0.990	65	0.065
MLVA-16	0.992	0.990–0.994	75	0.037

### MLVA genotyping results

**Figure 6.**
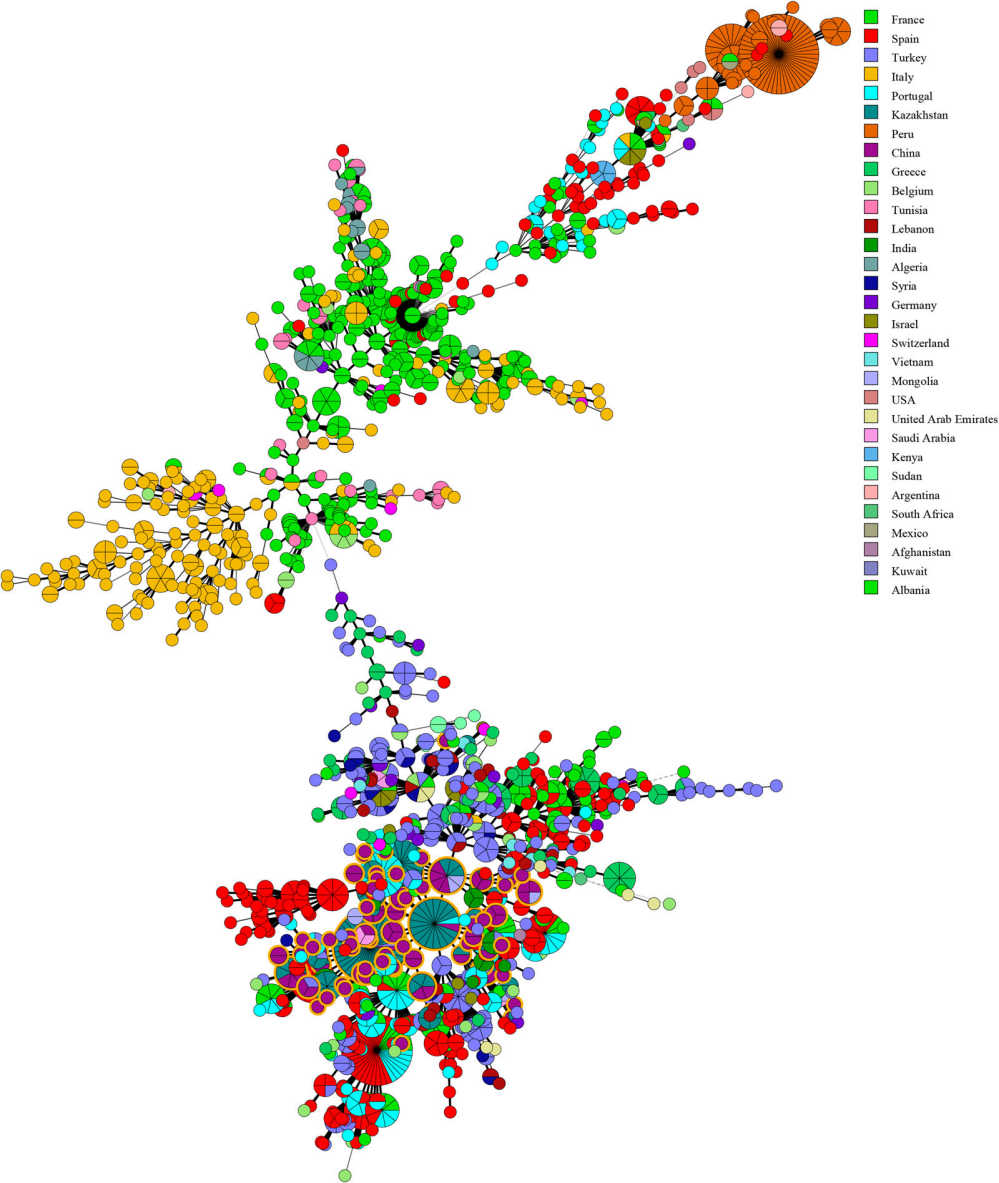
Minimum Spanning Tree (MST) including 107 of our *B. melitensis* isolates characterized in this study. The tree was constructed using MLVA-16 data from 2080 profiles of *B. melitensis* available in the MLVA international database. Nodes including isolates from this study are highlighted in yellow.

All isolates were further characterized by MLVA-16. The 107 *B. melitensis* isolates clustered into 75 genotypes, 54 of which were single genotypes represented by singular independent strains. The other 21 genotypes were shared by two to four isolates. With respect to panel 1, the *B. melitensis* population clustered into three MLVA-8 genotypes, including 42 (1-5-3-13-2-2-3-2; *n* = 76), 63 (1-5-3-13-2-3-3-2; *n* = 29), and one new genotype N1 (from MLVAbank v1.4.0) (1-5-3-13-2-4-3-2; *n* = 2). The clustering analysis indicated that the 107 *B. melitensis* isolates could be divided into seven main clusters with a genetic similarity coefficient ranging from 77% to 100%. Clusters A, B, C, E, and G contained genotype 42. Cluster D contained the two genotypes (42 and 63); moreover, Cluster F included the three genotypes (42, 63, and N1). A dendrogram of the 107 *B. melitensis* strains showed the strain identification results, panel 1 genotype, panel 1 + panel 2A genotype, their geographical source, and year of isolation (Figure 6).

In the present study, four different MLVA-11 genotypes were identified; three of these genotypes were previously described (116, 111, and 297), and the remaining one novel genotype (1-5-3-13-2-4-3-2-4-41-8) represented two strains with the single locus variant of MLVA-11 genotype 116 (1-5-3-13-2-2-3-2-4-41-8) and have not yet been assigned genotype (GT) numbers in the MLVA bank (http://microbesgenotyping.i2bc.paris-saclay.fr/). The new genotypes were found in the Tongliao area, and no other new GT was observed in this study. The most common genotypes, both MLVA-11 genotype 116 and 111, were the predominant genotypes and were distributed throughout all examined regions.

### MLVA-16 cluster analysis

In this study, MLVA was used to determine the molecular epidemiological links among *B. melitensis* strains (Table S1). Seven genotypes (GT 1, 30, 35, 36, 59, 61, and 65) were shared by strains from two different areas of Inner Mongolia, China, including Huhhot and Ulanqab, Hulun Buir and Huhhot, Alxa League and Hulun Buir, and Hinggan League and Tongliao. Fifteen genotypes (GT1, 9, 10, 17, 18, 21, 25, 26, 29, 37, 42, 50, 59, 63, and 70) were shared by two to four strains from the same areas. Each of the other 54 independent genotypes represented a single isolate. Moreover, both cluster branches A1 and B1 containing six and 18 strains were obtained from the Hulun Buir region, and the isolates from each branch shared identical or similar MLVA-16 genotypes (Figure 5).

### Global genetic relationship analysis of *B. melitensis* strains

In the present study, the genetic relationship of strains from Inner Mongolia, China and worldwide was investigated using MLVA-16. An MST was constructed on the basis of an analysis of the 107 MLVA-16 profiles of *B. melitensis* obtained from the current study and 2180 *B. melitensis* strains representing the three main lineages characterized as East Mediterranean, West Mediterranean, and American in the MLVA bank (2016, V1.4.0). The MST analysis clustered the considered strains into three large groups, and all strains from this study belonged to the East Mediterranean group. Some identical MLVA-16 profiles were found among strains from Mongolia, Kazakhstan, Portugal, and Turkey. In particular, many shared genotypes were observed between the strains from Inner Mongolia and Kazakhstan (Figure 6).

## Discussion

In the last 12 years, brucellosis has re-emerged in most regions of China, showing an annual increase in animal and human infections during this period [21,22]. The prevalence and dissemination of animals (sheep) brucellosis were very serious, resulting to extensive human brucellosis and incidence rate of human brucellosis in Inner Mongolia Autonomous Region was the ranked top three in Chinese provinces [19]. Strategies for prevention of animal brucellosis have changed since 2015 with vaccinations being a requirement in areas with high brucellosis prevalence. Animal infection was neglected due to difficulty in distinguishing immunity from natural infection [9]. Human disease epidemic data collection could reflect the animal brucellosis situation. The results showed that human brucellosis increased annually from 2012 to 2016, suggesting that animal brucellosis still developed in major parts of Inner Mongolia despite the use of vaccines for disease control. Therefore, prevention and control measures should be strengthened in the future.

Our investigation confirmed that seroprevalence in eastern regions were higher than central and western regions. This conclusion was closely related with reserves of small ruminants. Generally, Inner Mongolia iss divided into three geographical areas, including eastern, central, and western regions. A large number of farm animals are concentrated in the eastern and central regions, which were typical grassland areas. Small ruminant breeding is the main local economic source in these regions, especially in Hulun Buir which is a typical nomadic area, with poor sanitation and primitive living habits, leading to a large number of new brucellosis cases [23]. Western regions such as Bayannur City is located close to the Yellow River, and agriculture is the main economic industry, with the numbers of small ruminants lower than in other regions. Seroprevalence of human brucellosis exhibited an increasing trend from 2012 to 2016, but the incidence rate of human brucellosis showed a gradually declining trend. This result showed that when compared with previous years, new human brucellosis decreased gradually. In addition, a significant linear correlation (*r* = 0.627, *P* < 0.01) was observed between infected people and incidence rate of brucellosis. However, relatively large differences were presented among the 12 regions, suggesting that treatment and diagnostic routines maybe neglected and delayed in the regions where a low incidence of brucellosis occurs but a high number of infected persons are present [24]. Therefore, clinical diagnostic testing should be improved to strengthen public healthcare systems. However, Hohhot, a capital city of Inner Mongolia, has more *Brucella*-infected people and is also the most active area for animal trade and circulation in Inner Mongolia. There was no death cases in this study, mortality in cases of brucellosis is very low, certainly less than 5% and probably less than 2% [25,26].

Furthermore, brucellosis pathogens from 12 areas of the Inner Mongolia Autonomous Region were detected during 2012–2016. In all, 107 *Brucella* spp*.* strains were isolated from seven areas of the examined region. It was important to follow up on the control strategies and implement prevention measures for the identification and typing of *Brucella* isolates [27]. Subsequently, these strains were identified and typed using classical biotyping methods and molecular schedules. All isolates were identified as *B. melitensis*, the strain numbers of *B. melitensis* bv. 1, *B. melitensis* bv. 2, *B. melitensis bv. 3*, and *B. melitensis bv.* Var were 30, 2, 71, and 3, respectively. *Brucellus melitensis* bv. 3 was the predominant *Brucella* species in the examined region and accounted for a large number of human brucellosis cases. Our previous study reported that *B. melitensis bv. 3* is a sub-species that leads to a relatively high incidence of brucellosis in the Ulanqab region [19]; this species has also been responsible for most human brucellosis cases in China and worldwide [28]. A comprehensive control plan of sheep brucellosis should be implemented in this region, including continuous vaccination, limiting animal transfer of those suspected to have the disease and elimination of infected animals (sheep).

Both the discrimination ability of MLVA-16 and genetic diversity of the 107 *B. melitensis* strains were investigated as a part of this study. Many studies indicated that the MLVA-16 schedule had a higher resolution for *Brucella* spp*.*, despite the fact that *B. melitensi*s strains have relatively high genetic homogeneity. The polymorphism index of MLVA-16 was 0.992 on the basis of the HGDI, of which bruce04, bruce16, and bruce30, the three loci in panel 2B, were the most useful for genotyping analysis of isolates from this region. In Asian countries including China, bruce07 and bruce09 in panel 2B loci consistently showed low HGDI values. In most European countries, all loci in panel 2B were highly discriminatory [29]. These data suggested that mutants occurred only in a high (panel 2B) variety of loci, and the pathogens considered in this study might have originated from a recent common ancestor.

MLVA-8 genotypes 42 and 63 were the most common genotypes in China and other developing countries with a relatively high incidence of brucellosis; in particular, MLVA-8 genotype 42 was observed to be the most common genotype worldwide and had an important significant for epidemiology of human and animal brucellosis [30]. MLVA-8 genotype N1 is a single-locus variant (SLV) of genotype 63 and belongs to the group of novel genotypes when compared with the strains from MLVAbank v1.4.0. The two strains showed a new genotype, which was isolated from Tongliao, Inner Mongolia Autonomous Region; the two isolates were from the same time and shared extremely similar MLVA-16 genotypes. These data suggested that the two strains may only circulate in the small but wide Tongliao area and have an epidemiology significant of this novel genotype, which needs to be examined further. The incidence rate of brucellosis in the Tongliao area ranked in the top five in the Inner Mongolia Autonomous Region. Moreover, we could not find any data about the molecular epidemiological characteristics of the isolates from this area; therefore, enhanced pathogen surveillance and a molecular epidemiology investigation of field strains should be implemented to reveal epidemiology characteristics of brucellosis in this region.

In the present study, MLVA-11 schedule was used for the geological origin analysis of this population; all of the isolates belonged to the East Mediterranean group. MLVA-11 genotype 116, representing 70% (75/107) of isolates, was widely distributed throughout most of China. The data analysis revealed two MLVA-11 genotypes (116 and 111) in the Hulun Buir area. Moreover, isolates from Huhhot and Ulanqab were dominated by MLVA-11 genotype 116, and isolates from Alxa League and Hinggan League belonged to MLVA-11 genotype 111. Previous studies have reported that MLVA-11 was widely used for tracing the geographical origin of the source of infection [31]. These data hinted that isolates from different geological areas of the Inner Mongolia region exhibited potential origin diversity; one probable explanation for this is strains with a common ancestor but different evolution paths. An in-depth molecular analysis of more isolates should be conducted future to further examine the geological distribution and historical origin of isolates from Inner Mongolia. Notably, isolates obtained from the Tongliao area formed three types of MLVA-11 genotypes, including 116, 111, and CN1 genotypes. Although fewer strains were isolated in this study, this information implied that the isolate origin was complex and requires in-depth analysis.

A complete MLVA-16 assay is often used to trace-back the source of infections and investigate molecular epidemiological links among *Brucella* isolates. In this study, the molecular epidemiological relationships of 107 *B. melitensis* strains were investigated. Five genotypes (GT 1, 35, 36, 59 61, and 65) containing strains from two different areas shared an identical MLVA-16 genotype; these pairs of areas included Huhhot and Ulanqab, Hulun Buir and Huhhot, Alxa League and Hulun Buir, and Hinggan League and Tongliao. Many studies have confirmed that the MLVA-16 genotyping results correlate well with the epidemiological data of epidemiologically related isolates displaying identical or closely related genotypes [32]. These data suggested that these regions contained cross-infections cases, and the disorder transfer from infected animals was one of the main reasons for the same. In addition, 15 shared genotypes (shadow genotypes) observed during this analysis were among the 40 *B. melitensis* strains obtained from the same area. This information suggested the occurrence of a multipoint outbreak epidemic from multiple common sources. Each of the 54 independence genotypes represented a unique strain that showed sporadic or no epidemiology links among brucellosis cases. Cluster branches A1 and B1 consisted of six and 18 strains from the Hulun Buir region, respectively, and strains from each branch shared identical or similar genotypes. Furthermore, MLVA genotyping confirmed the occurrence of a relatively large-scale human brucellosis outbreak in this region, caused by both *B. melitensis bv. 1 and bv. 3*. Our previous study verified that the prevalence of brucellosis in Ulanqab significantly correlated (*r* = 0.627, *P* < 0.01) with brucellosis morbidity of the neighbouring provinces [19]. Due to an imbalance in the number of samples among different areas, in order to avoid over-interpreting our data, we did not compare our strains with those of other provinces in China. A large sample of humans and animals should be analyzed to further elucidate the genetic diversity and epidemiological characteristics of *Brucella* spp*.* in the Inner Mongolia Autonomous Region.

The Inner Mongolia Autonomous Region has the highest incidence rate of human and animal brucellosis throughout China, and many *Brucella species* were isolated and analyzed. In order to better understand the genetic relationships and historical origin of strains considered in this study, we used the MLVA-16 schedule for molecular investigation on a global scale. As expected, a few identical MLVA-16 profiles were found between strains from this study and strains from Mongolia, Kazakhstan, and Portugal. In particular, many shared genotypes were observed between the strains from China and Kazakhstan. We believe that this conclusion is consistent with the epidemiology of brucellosis between the two countries. Kazakhstan and China have had a long-term trade partnership: there is a long exchange and trade history of animals and its products between the two nations [33]. Moreover, in the past few centuries, livestock exchange was the main payment method for nomadic people between China and Kazakhstan in the past few centuries [34]. Furthermore, animal introduction based on official or nongovernmental reasons occurs frequently, leading to persistent circulation of strains in these regions. A further analysis of these strains using next-generation sequencing would be helpful to better understand the epidemiology of human and animal brucellosis in China and its neighbouring areas [35]. Moreover, our study has some limitations. First, the data used were collected from a large geographical area that might have been influenced by laboratory tests and equipment conditions, or the physician’s understanding of the disease and local economic development status. Second, due to variability in the number of serum samples and strains collected among different regions and for different years, there may be some deviation in the conclusion. Thus, future research with additional samples in some regions is essential. Third, the study data were not reported before 2012 or after 2016; therefore, epidemiology characteristics and relatedness of the strains could only be analyzed between 2012 and 2016.

## Conclusions

In the present study, a comprehensive investigation of epidemiology characteristics in Inner Mongolia was performed. A total of 1102304 serum samples were collected for testing, and a 3.79% positive rate of anti-*Brucella* was found. The average positive rates had a large imbalance from 0.90 to 7.07% in the 12 regions examined, and a significant linear correlation between the number of infected people and the incidence rate was observed. Moreover, there were at least four *Brucella* (*B. melitensis* 1, 2, 3, and variants) species in the examined region. The epidemic characteristics of human brucellosis in this area were revealed from the perspective of molecular genetics, involved in cross-infection, outbreak (multi-point), and sporadic occurrences. In addition, there was a broad genetic relationship among the strains from Hulun Buir, confirming a serious human brucellosis epidemic in this region. These data suggested that the disordered transfer from infected animals and an illegal exchange of animals led to the spread of infection among animals and humans. Therefore, etiological surveillance should be strengthened in the entire examined area. Furthermore, a few identical MLVA-16 profiles were found between strains obtained from this study and its neighbours, Mongolia and Kazakhstan, indicating that these strains exhibited a high genetic homogeny and had a common geographical origin.

## Supplementary Material

Supplemental Material
